# Cultural and spiritual acumen in Chinese urology nursing: a quantitative and qualitative pilot investigation

**DOI:** 10.3389/fmed.2025.1666924

**Published:** 2025-11-24

**Authors:** Wenting Zhu, Yilin Wang, Minjia Gu, Liqing Zheng, Qin Yan, Dongmei Ren

**Affiliations:** 1 Department of Urology, Jiading District Central Hospital Affiliated Shanghai University of Medicine and Health Sciences, Shanghai, China; 2 Department of Gynaecology and Obstetrics, Jiading District Central Hospital Affiliated Shanghai University of Medicine and Health Sciences, Shanghai, China; 3 Department of Nursing, Jiading District Central Hospital Affiliated Shanghai University of Medicine and Health Sciences, Shanghai, China

**Keywords:** cultural care competencies, nurses, professional characteristics, spiritual care competencies, urology

## Abstract

**Background:**

While the importance of cultural and spiritual care proficiencies is well-validated in cancer supportive care and critical care settings, their relevance in urology remains to be substantiated. Gauging the spiritual care requisites of nurses within urology divisions may foster enhanced sentiments of hope, serenity, and fortitude among patients.

**Objectives:**

This study aimed to examine cultural and spiritual care proficiencies among nurses tending to urology patients and to ascertain the correlation between the personal and professional attributes of these nurses and their perspectives on cultural and spiritual care competencies.

**Methods:**

Thirty-five nurses affiliated with the urology department of Jiading District Central Hospital, Shanghai University of Medicine and Health Sciences, China, were provided with a questionnaire encompassing the spiritual care competency scale and the cultural competence scale. All participating nurse practitioners were female, of Han Chinese ethnicity, adhered to Chinese culture, and were devoid of religious affiliations. Both instruments used a 6-point Likert scale (1: vehemently disagree, 2: disagree, 3: slightly disagree, 4: slightly agree, 5: agree, and 6: vehemently agree).

**Results:**

Participating nurses possessed education at or above the junior college level. Nurses aged 35 and older accounted for 26%, while 63% had a decade or fewer of experience in urological nursing in China. The preeminent value within the spiritual care competency scale pertained to spiritual perspectives (factor 2; 4.94 ± 0.32/nurse), succeeded by attributes for spiritual care (factor 1; 4.42 ± 0.19/nurse), defining spiritual care (factor 3; 4.33 ± 0.21/nurse), spiritual care attitudes (factor 4; 4.2 ± 0.28/nurse), spiritual care values (factor 5; 3.98 ± 0.21/nurse), and spiritual care personal values (factor 6; 3.89 ± 0.22/nurse). The clinical nursing cultural competence scale registered at 4.24 ± 0.79/nurse. No associations were evident between personal/professional traits and perceptions of cultural and spiritual care competencies (*p* > 0.05 for all comparisons).

**Conclusion:**

Nurses functioning within the urology department exhibited an affirmative outlook toward cultural and spiritual care proficiencies. The personal and professional attributes of nurses in urology are generally uncorrelated with their perceptions of cultural and spiritual care competencies. Nurses in the urology department have a heavy workload and insufficient knowledge of spiritual care practices.

## Introduction

Spiritual care, predicated on astute attention to patients’ values and beliefs, demands considerable expertise and is enacted within a relationship of trust and confidence between healthcare professionals and patients (1). Spirituality constitutes an indispensable element of wellbeing, particularly for patients (2). Spiritual care identifies and addresses the exigencies of individuals confronting ill health, trauma, and life crises (3), correlating to enhanced quality of life and salutary health outcomes (4). The term ‘spiritus’, derived from the Latin for ‘to breathe’, denotes the vital essence of life, the wellspring of spirituality (5). China’s Health Planning Commission has affirmed the efficacy of cultural and spiritual care in improving the health outcomes of individuals experiencing ill health, trauma, and life crises (6), while underscoring the imperative for heightened cultural competence awareness among Chinese clinical nurses (7). Disparities persist in the competence and perceptions of spiritual care among these nurses (8). The significance of cultural and spiritual care competencies is well-documented in cancer supportive care (5, 9) and critical care units (7). Comparative analyses of these competencies across diverse diseases and settings are detailed in Table 1. However, their role remains nascent in urology (10), despite their manifest importance for nurses attending to urological patients (11). Ascertaining the spiritual needs of nurses within urology departments may foster hope, tranquility, and resilience in patients, encourage positive health behaviors, and facilitate the selection of appropriate coping mechanisms (10). Existing research on spirituality has predominantly focused on populations within England and the United States, largely encompassing Christian cultures (2), thereby accentuating the need for further investigation into spirituality in nursing across diverse ethnicities within the Asian populace (12).

**Table 1:** Details of comparative studies on cultural and spiritual care competencies for different diseases in different settings.

Study	Published year	Subject ethnicity	Sample size (N)	Age (years)	The subject associated with conditions	Main conclusions
Group concept mapping, Hvidt et al. (1)	2020	Danish	43	27–75	Investigators, scholars, and practitioners	Attending to patients’ values and beliefs within spiritual care mandates commensurate expertise.
Cross-sectional study, Chew et al. (2)	2016	Singaporean	1,008	>18	Nursing staff	Nurses practicing within the acute care setting exhibited a propitious disposition concerning spirituality and the provision of spiritual care.
Integrative research review, Counted et al. (4)	2018	Real-world	132,053	>18	Individual, whether healthy or afflicted	Experiencing a sense of interconnectedness seems to augur well for one’s health.
Descriptive cross-sectional study, Wang et al. (5)	2024	Chinese	357	>18	Metastatic breast carcinoma in women	Chinese women confronting advanced breast cancer require refined spiritual succor.
A cross-sectional survey, Wang et al. (7)	2022	Chinese	3,858	>18	Clinical nurses	Within the Chinese healthcare paradigm, the augmentation of cultural competency among clinical nurses is deemed imperative.
A multicenter cross-sectional study, Guo et al. (8)	2024	Chinese	1,227	>18	Clinical nurses	Chinese clinical nurses exhibit varying levels of competence in, and perceptions of, spiritual care.
A cross-sectional study, Cheng et al. (9)	2019	Chinese	173	>18	Post-surgical breast cancer patients undergoing chemotherapeutic treatment	Bespoke spiritual support is paramount for postoperative breast cancer patients undergoing chemotherapy.
A cross-sectional descriptive study, Özveren et al. (10)	2022	Turkish	220	>18	Urinatingly challenged patients	Individuals grappling with urinary incontinence necessitate spiritual succor.

Non-categorical variables are depicted as median (Q3–Q1).

## Objectives

This cross-sectional pilot study aimed to investigate the cultural and spiritual care competencies possessed by nurses tending to urology patients. Furthermore, it aimed to assess the correlation between nurses’ personal and professional attributes and their perceived cultural and spiritual care competencies within the urological context.

## Materials and methods

### Ethics approval and consent to participate

The authors meticulously crafted the cross-sectional study’s protocols, subsequently ratified by the Human Ethics Committee of Jiading District Central Hospital, Shanghai University of Medicine and Health Sciences (Approval No. SJdcH14178, dated December 5, 2022). This investigation adheres strictly to Chinese law and the v2008 Declaration of Helsinki (latest Chinese iteration). Prior to commencement, informed consent for participation and publication (across diverse formats and publications) was secured from all participating nurses. Ethical endorsement encompassed the authorized utilization of instruments—the Spiritual Care Competency Scale (Chinese version) and the Clinical Nursing Cultural Competence Scale (Chinese version)—through approvals granted by both the institutional Human Ethics Committee and the Department of Health and Education of China.

### Design and setting

A quantitative, subjective, pilot cross-sectional study was conducted among nurses providing care to urology patients at Jiading District Central Hospital, Shanghai University of Medicine and Health Sciences, China (a secondary-level hospital without affiliation to a tertiary institution).

### Inclusion criteria

The study encompassed all nursing staff affiliated with urological patients, specifically those serving within the urology division from 31 December 2022 to 31 December 2024.

### Exclusion criteria

Nurses declining participation by withholding data were excluded from the study; non-respondents were neither tracked nor comparatively analyzed.

### Sample size calculations

Using a descriptive methodology with Kendall’s and Spearman’s correlation analyses (13), predicated on the questionnaire volume, and stipulating *α* = 0.05, *β* = 0.8, a 95% confidence interval (CI), and 2.3 participants per variable, the calculated sample size for this study comprised 35 nurses.

## Outcome measures

### The spiritual care competency scale (Chinese version)

Comprising 27 questions in total, responses are gauged via a 6-point Likert scale (encompassing vehemently disagree, slightly disagree, disagree, vehemently agree, slightly agree, and agree). The spiritual care competency scale exhibits a six-factor structure, elaborated upon in Table 2. Cronbach’s *α* values range from 0.71 to 0.82 (14).

**Table 2:** Six-factor model for the Chinese version of the spiritual care competency scale.

Factor	The component of the spiritual care
1	Attributes for spiritual care
2	Spiritual perspectives
3	Defining spiritual care
4	Spiritual care attitudes
5	Spiritual care values
6	Spiritual care and personal values

### The clinical nursing cultural competence scale (Chinese version)

Cultural competence was delineated into four facets, gauged via a 29-item instrument. These items encompass cultural proficiencies (11 items), cultural erudition (8 items), cultural acumen (7 items), and cultural sensitivity (3 items). Responses are predicated on a 6-point Likert scale (ranging from vehement disagreement to tempered agreement). The derived Cronbach’s *α* was 0.88 (15).

### Scale measurements and data collections

Each nurse was furnished with a QR-coded questionnaire to be duly completed within 1 week. After completion, the researchers shall collate the data in the background.

A 6-point Likert scale, as delineated in Table 3, was used for both instruments: the spiritual care competency scale and the clinical nursing cultural competence scale. Both instruments have been validated for use in Chinese populations. The authors personally administered both questionnaires and conducted the scoring process.

**Table 3:** Six-point Likert scale for the Chinese version of the spiritual care competency scale and the Chinese version of the clinical nursing cultural competence scale.

Likert scale	Corresponding point
Vehemently disagree	1
Disagree	2
Slightly disagree	3
Slightly agree	4
Agree	5
Vehemently agree	6

Scoring was done by the authors themselves.

## Data analysis

Statistical analyses were conducted utilizing InStat 3.01 (GraphPad Software, San Diego, CA, USA). Categorical variables are presented as frequencies (percentages). Continuous, normally distributed variables are summarized as mean ± standard deviation (SD). Non-normally distributed continuous variables are expressed as median [interquartile range (Q3–Q1)]. Means and SD were used for normal distributions; otherwise, median and quartiles (Q1, Q3) were reported when n was > 8; if n was ≤ 8, the Min-Max range was utilized. The Soup calculator® LLB (USA) was used for median, Q3, and Q1 calculations. The chi-square (*ꭓ*^2^) or Fisher’s exact test was applied to categorical variables. The Kolmogorov–Smirnov test assessed continuous variable normality, while the Bartlett test evaluated variance homogeneity in normally distributed continuous variables. The Mann–Whitney *U*-tests were used for univariate analyses. Both univariate and subsequent multivariate (logistic regression) analyses assessed associations between personal/professional characteristics of urology nurses and perceptions of cultural/spiritual care competencies (2). Significance was defined as a *p*-value of < 0.05 at 95% CI (two-tailed). Forest and funnel plots were generated via metaanalysisonline.com (2024–2025, ELIXIR Hungary). *I*^2^ > 50% indicated moderate heterogeneity. VisualGPT’s map maker^1^ was used to generate flowcharts.^2^ AI generator created the Graphical Abstract. Premium English Translator^3^ and Grammarly enhanced the English language. The Sentence Shortener^4^ AI condensed the text.

## Results

### Cohort demographics

**Figure 1:**
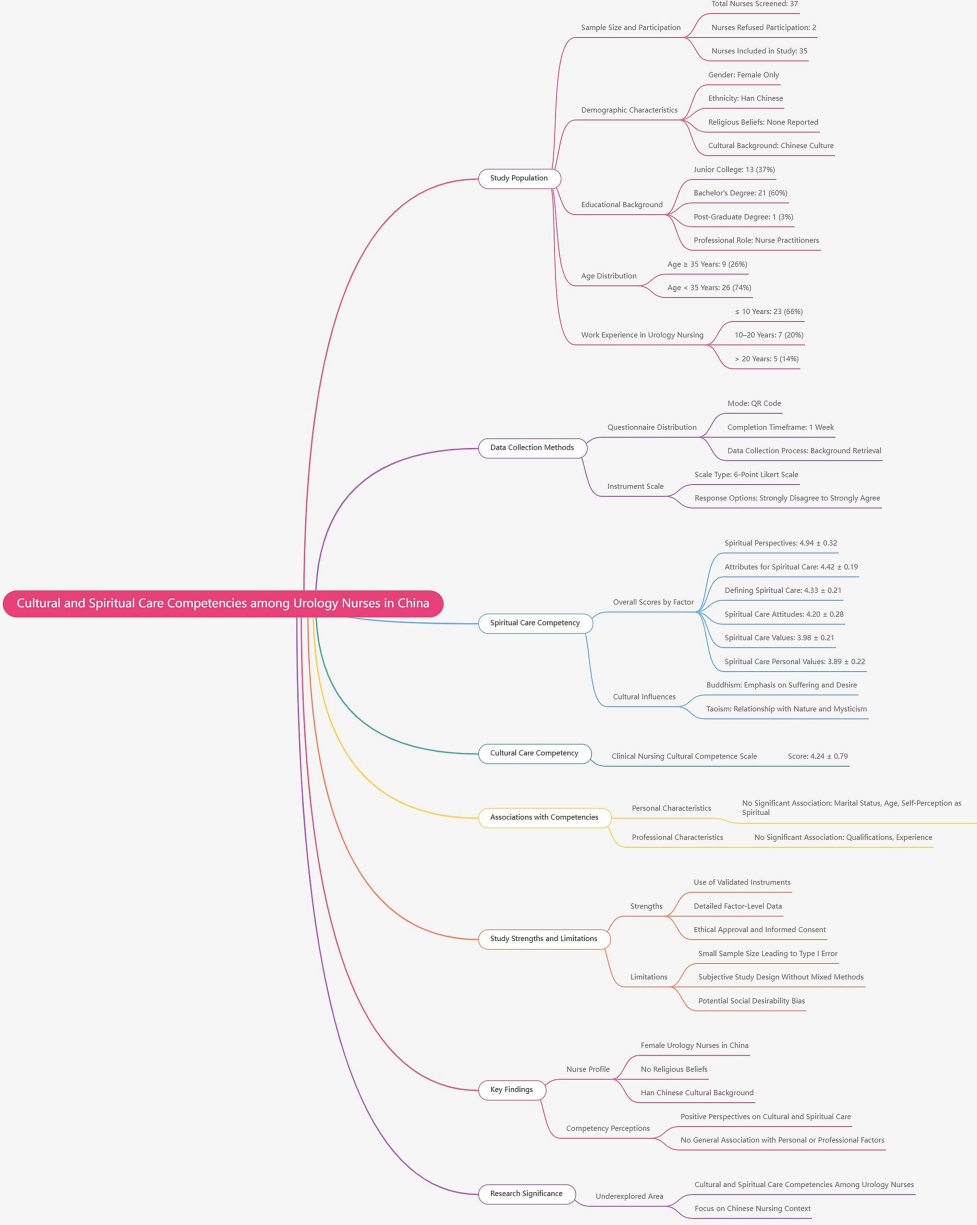
Flowchart delineates the cross-sectional study design.

From 31 December 2022 to 31 December 2024, a cohort of 37 nurses underwent screening for study eligibility. Of these, two declined to furnish comprehensive data, resulting in their exclusion (*n* = 2). Data originating from 35 nurses used within the urology department of Jiading District Central Hospital, Shanghai University of Medicine and Health Sciences, Shanghai, China, were included in the study. A flow diagram illustrating the cross-sectional pilot study design is provided in Figure 1.

## Outcome measures

### Nurse demographics

The cohort solely comprised individuals identifying as female, with no professed religious affiliations. All participants self-identified as being of Han Chinese ethnicity, possessed no less than a junior college-level education, and were registered nurse practitioners, demonstrably aligned with prevailing Chinese cultural norms. Nurses aged 35 years and older accounted for 26%, while 66% had a decade or fewer of experience in urological nursing within China. Comprehensive demographic and professional data pertaining to nurses engaged in urological patient care are delineated in Table 4.

**Table 4:** Personal and professional characteristics of the nurses caring for urology patients.

Characteristics	Value
Number of nurses	35
Age (years)	
Under 35 years	26(74)
35 years and above	9(26)
Working experience (years)^a^	
More than 20 years	5(14)
10 years to 20 years	7(20)
10 years or less	23(66)
Marital status	
Married	17(49)
Single	18(51)
Education	
Bachelor’s degree	21(60)
Post-graduate degree	1(3)
Junior college	13(37)
Professional title	
Nurse practitioner	20(57)
Primary nurse	8(23)
Nurse-in-charge	7(20)
Deputy director nurse	0(0)
Perceiving self as spiritual	
Yes	30(86)
No	5(14)

a Experience of urology nursing care in China.

Variables are expressed as frequencies with percentages in parentheses.

### Spiritual care competency scale (Chinese version)

Factor 2 exhibited the highest values, followed sequentially by factors 1, 3, 4, 5, and 6. Granular data about the Spiritual Care Competency Scale are presented in Table 5.

**Table 5:** Details of the Chinese version of the spiritual care competency scale for nurses.

Parameters	The Chinese version of the spiritual care competency scale					
	Factor 1	Factor 2	Factor 3	Factor 4	Factor 5	Factor 6	Parameter	Attributes for spiritual care	Spiritual perspectives	Defining spiritual care	Spiritual care attitudes	Spiritual care values	Spiritual care and personal values
Value/ nurse	4.42 ± 0.19	4.94 ± 0.32	4.33 ± 0.21	4.2 ± 0.28	3.98 ± 0.21	3.89 ± 0.22

Value presented as mean ± standard deviation (SD).

1: vehemently disagree, 2: disagree, 3: slightly disagree, 4: slightly agree, 5: agree, and 6: vehemently agree.

Number of nurses: 35.

Number of questions: 27 (Chinese language sheet; we do not have permission to publish a list of questions; institutional integrity).

### Clinical nursing cultural competence scale (Chinese version)

The Clinical Nursing Cultural Competence Scale yielded a mean score of 4.24 ± 0.79 per nurse (Table 6).

**Table 6:** Details of the Chinese version of the clinical nursing cultural competence scale.

Parameter	Value
Number of nurses	35
Scale/ nurse	4.24 ± 0.79

Value presented as mean ± standard deviation (SD).

1: vehemently disagree, 2: disagree, 3: slightly disagree, 4: slightly agree, 5: agree, and 6: vehemently agree.

Number of questions: 29 (Chinese language sheet; we do not have permission to publish the list of questions; institutional integrity).

### Correlation analysis

A univariate analysis identified marital status (*p* = 0.045, Mann–Whitney U-test), age ≥ 35 years (*p* = 0.039, Mann–Whitney U-test), and self-perceived spirituality (*p* = 0.041, Mann–Whitney U-test) as factors correlated with perceptions of cultural and spiritual care competencies (univariate analysis data not included). Multivariate logistic regression analyses revealed no statistically significant associations between personal/professional characteristics and perceived cultural/spiritual care competencies. The specifics are detailed in Table 7.

**Table 7:** Multivariate logistic regression analyses for the association between personal and professional characteristics of nurses and perceptions of cultural and spiritual care competencies.

Parameter	*p*-value	Odds ratio	95% CI
Marital status (married vs. single)	0.0521	0.9841	0.9121–0.9982
Age (≥35 years vs. <35 years)	0.0532	0.842	0.8124–0.9251
Perceiving self as spiritual (yes vs. no)	0.0621	0.7783	0.7124–0.8521

A *p*-value less than 0.05 and an odds ratio greater than 1 are considered significant.

CI, confidence interval.

**Figure 2:**
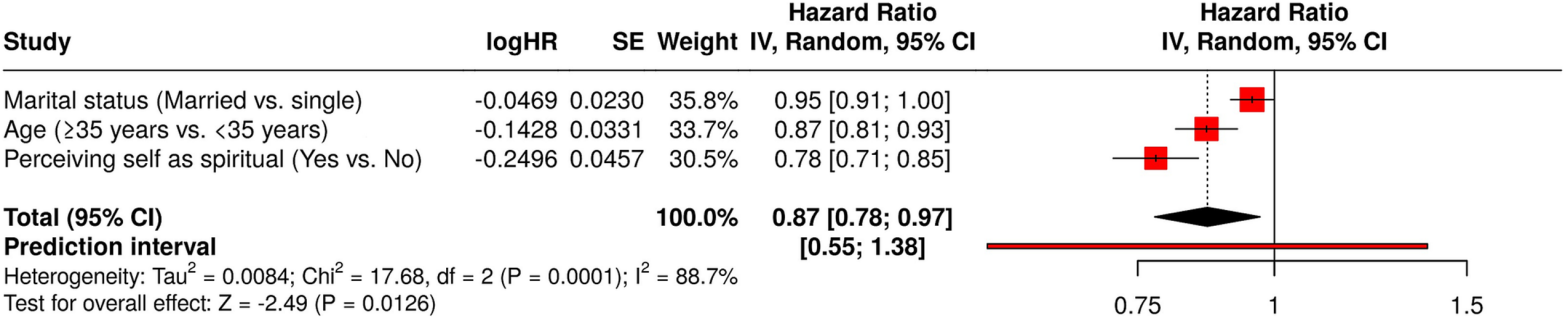
Forest plot illustrates the correlation between individual and occupational attributes and perspectives on cultural and spiritual care proficiencies.

Subsequent analysis incorporating three personal/professional characteristics (Figure 2), utilizing a random-effects model with inverse variance weighting to compute the hazard ratio (HR), revealed a statistically notable difference. The pooled HR was 0.87 (95% CI: 0.78–0.97; exact *p*-value for random effect was 0.013 and *t*-value was −2.49). The test for overall effect exhibited statistical significance (*p* < 0.05, random effects model with inverse variance). Significant heterogeneity was detected (*p* < 0.01, random effects model with inverse variance), implying variability in the magnitude and/or direction of individual personal/professional characteristics. The *I*^2^ statistic indicated that 88.7% of observed variance was attributed to heterogeneity rather than random error.

### Bias assessment

**Figure 3:**
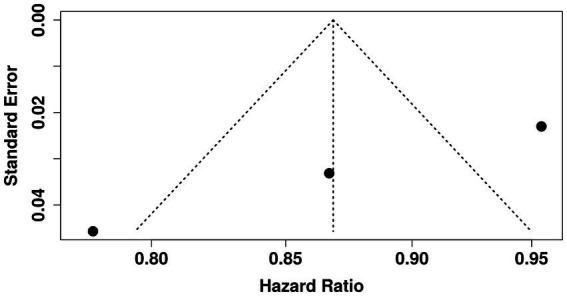
Funnel plot shows the association between personal and professional characteristics and perceptions of cultural and spiritual care competencies.

The funnel plot analysis (Figure 3) did not indicate substantial bias in nurse personal/professional characteristic assessment, though Egger’s test suggested funnel plot asymmetry (intercept: −9.03, 95% CI: −9.58 to −8.48, t: −32.22, *p*-value: 0.052, Egger’s test).

### Assumption testing

Details regarding the assumption tests conducted in this study, inclusive of justifications for the choice of the conclusive tests, are shown in Table 8.

**Table 8:** Results of assumption tests used in the study, with reasons for a conclusive test.

Parameters	Test
Kolmogorov and Smirnov method	Categorical, normally distributed continuous (in Kolmogorov and Smirnov method *p* > 0.05), and non-normally distributed continuous variables (in Kolmogorov and Smirnov method *p* < 0.05) are expressed as frequencies with percentages in parentheses, mean ± standard deviations (SD), and median with Q3–Q1 in parentheses, respectively.
Personal and professional characteristics of the nurses	
Working experience	Failed in normality test, *p* > 0.01. Normal continuous variable.
Univariate analysis between personal and professional characteristics of nurses and perceptions of cultural and spiritual care competencies	Not pass in normality test for marital status, age, and perceiving self as spiritual. A *p-*value was <0.05 for all. Therefore, a Mann–Whitney U-test was used.
Multivariate analysis	Logistic regression analysis was used.
Forest plot and funnel plot	Setting: random effects model, method: inverse, summary measures: standard mean difference, between study heterogeneity estimator: DerSimonian-Laird

## Discussion

All nurses are ethnically Han Chinese, female, and profess no religious affiliation. The gender demographics in this study align with the China Health Statistical Yearbook data from 2002 to 2020 (16), reflecting the higher proportion of women in Chinese healthcare. While China’s predominant ethnicity is Han Chinese (17), the ruling party maintains a secular stance (18). Consequently, nurses caring for urology patients in China are typically female, of Han Chinese ethnicity, and non-religious.

The spiritual care competency scale across all factors registered a mean score of 4 or higher per nurse (indicating mild agreement), while the cultural competence scale yielded a mean score of 4.24 ± 0.79 per nurse (surpassing mild agreement). These findings regarding spiritual care competency align with previous research, including a cross-sectional study (4.92 for acute care nurses’ spiritual care competency) (2), an integrative research review (moderate level for clinical nurses’ spiritual care competency) (4), a multicenter cross-sectional study (> 4 for clinical nurses’ spiritual care competency) (8), and a cross-sectional survey (moderate level for clinical nurses’ spiritual care competency) (7). In essence, nurses in Chinese urology departments exhibit a favorable disposition toward cultural and spiritual care competencies, which should be leveraged to cultivate stronger nurse–patient rapport.

Advanced age (≥ 35 years) and a self-perceived spiritual inclination, while not directly correlated with perceived cultural and spiritual care competence, emerge as salient variables in univariate analyses. These findings regarding the association between perceived cultural and spiritual care competence align with those of a prior cross-sectional study (7). Spiritual care transcends specific religious (e.g., Buddhist) or regional practices (1), demanding experiential knowledge and specialized expertise (1, 19). Spiritual care prioritizes an individual’s core beliefs and meaning-making, whereas cultural care honors their traditions, values, and customs (20). The personal and professional attributes of nurses in urology are generally independent of perceived cultural and spiritual care competencies, as such care constitutes a fundamental dimension of human existence (1).

The research, while termed a cross-sectional survey, incorporates a mere 35 nurses from a solitary medical facility. This markedly diminutive, homogenous, and unrepresentative cohort—comprising solely Han Chinese women, devoid of religious affiliations—substantially curtails the capacity for broad extrapolation. Given the extensive diversity among urology nurses nationwide, exemplified by our affiliation with Jiading District Central Hospital, Shanghai University of Medicine and Health Sciences, Shanghai, China, a sample of 35 cannot be deemed reflective of the Chinese nursing population at large. Acknowledging this limitation, the interpretation confines itself to the cohort under scrutiny, refraining from generalizations applicable to all nurses in China. Furthermore, an excessively large sample size may introduce unwarranted complexity and financial burdens, thereby rendering the study impracticable. Such circumstances constitute ethically untenable scenarios and warrant avoidance (21).

The mean scores for spiritual care and personal values registered below 4 (mild agreement), attributable to nurses’ misinterpretations of spiritual care or its conflation with religious perspectives (2). Conversely, spiritual perspective values exhibited higher mean scores across all factors, a finding resonant with the prevalence of Buddhism (with its pronounced emphasis on suffering rooted in desire) and Taoism (which emphasize the individual’s harmonious integration with nature through mystical insight, intuition, and acceptance of natural processes) in China (22). Moreover, spiritual care values also averaged below 4 (mild agreement), owing to nurses’ workload and insufficient knowledge of spiritual care practices (2). Consequently, authorities should prioritize addressing nurses’ perceptions of cultural and spiritual care competencies and implementing specialized training programs for urology nurses in these critical domains.

Following multivariate (logistic regression) analyses, univariate analyses were performed to ascertain the relationship between the individual and occupational characteristics of nurses caring for urology patients and their adjudged cultural and spiritual care aptitudes. For this study, forest and funnel plots using a random-effects model with the inverse variance method were preferred. Employing the univariate analysis as a predictive ‘filter’ within a multivariable model is erroneous (23). The employment of both univariate and multivariable regression requires justification, given their potential to yield contradictory outcomes (23). Furthermore, concurrent model usage cannot be considered a validation ‘instrument’; a singular approach is thus advisable. Therefore, this study exclusively utilized forest and funnel plots, leveraging a random-effects model with the inverse variance method, to examine the association between nurses’ personal and professional attributes and their perceived cultural and spiritual care expertise in urology patient management.

## Limitations

Despite the study’s commendable focus on an under-researched demographic (urology nurses in China), utilization of validated instruments for spiritual and cultural care competency assessment, provision of granular factor-level data conducive to targeted training initiatives, and adherence to rigorous ethical approval and informed consent protocols, several limitations warrant consideration. The modest sample size elevates the risk of Type I error, and the study’s reliance on subjective methodologies, without an objective mixed-methods design (1), introduces potential bias from social desirability responses (2). Beyond age and educational attainment, other examined variables demonstrated negligible influence on the perceptions of cultural and spiritual care competencies. The sample’s homogeneity restricts the generalizability of findings beyond the immediate study context, and the conclusions drawn should be regarded as preliminary.

## Conclusion

Urology nurses demonstrated a salutary outlook on cultural and spiritual care proficiencies. Personal and professional attributes of urology nurses are generally dissociated from perceptions of cultural and spiritual care competence. Authorities should prioritize nurses’ perceptions regarding cultural and spiritual care aptitude and the provision of specialized training in these domains. This study addresses a hitherto underexplored subject: cultural and spiritual care competence among urology nurses in China.

## Data Availability

The original contributions presented in the study are included in the article/supplementary material, further inquiries can be directed to the corresponding author.

## Footnotes

^1^ https://visualgpt.io/mind-map-maker

^2^ https://viscript.app/sign_up

^3^ https://anythingtranslate.com/translators/premium-english-translator/

^4^ https://www.junia.ai/tools/sentence-shortener
